# Learning in real world practice: Identifying implementation strategies to integrate health-related social needs screening within a large health system

**DOI:** 10.1017/cts.2023.652

**Published:** 2023-10-16

**Authors:** Kevin Fiori, Samantha Levano, Jessica Haughton, Renee Whiskey-LaLanne, Andrew Telzak, Sybil Hodgson, Elizabeth Spurrell-Huss, Allison Stark

**Affiliations:** 1 Department of Family & Social Medicine, Albert Einstein College of Medicine, Bronx, NY, USA; 2 Department of Pediatrics, Albert Einstein College of Medicine, Bronx, NY, USA; 3 Office of Community & Population Health, Montefiore Health System, Bronx, NY, USA; 4 Montefiore Medical Group, Bronx, NY, USA; 5 Department of Medicine, Albert Einstein College of Medicine, Bronx, NY, USA

**Keywords:** Social determinants of health, health-related social needs, health equity, learning health system, quality improvement

## Abstract

**Introduction:**

Health systems have many incentives to screen patients for health-related social needs (HRSNs) due to growing evidence that social determinants of health impact outcomes and a new regulatory context that requires health equity measures. This study describes the experience of one large urban health system in scaling HRSN screening by implementing improvement strategies over five years, from 2018 to 2023.

**Methods:**

In 2018, the health system adapted a 10-item HRSN screening tool from a widely used, validated instrument. Implementation strategies aimed to foster screening were retrospectively reviewed and categorized according to the Expert Recommendations for Implementing Change (ERIC) study. Statistical process control methods were utilized to determine whether implementation strategies contributed to improvements in HRSN screening activities.

**Results:**

There were 280,757 HRSN screens administered across 311 clinical teams in the health system between April 2018 and March 2023. Implementation strategies linked to increased screening included integrating screening within an online patient portal (ERIC strategy: involve patients/consumers and family members), expansion to discrete clinical teams (ERIC strategy: change service sites), providing data feedback loops (ERIC strategy: facilitate relay of clinical data to providers), and deploying Community Health Workers to address HRSNs (ERIC strategy: create new clinical teams).

**Conclusion:**

Implementation strategies designed to promote efficiency, foster universal screening, link patients to resources, and provide clinical teams with an easy-to-integrate tool appear to have the greatest impact on HRSN screening uptake. Sustained increases in screening demonstrate the cumulative effects of implementation strategies and the health system’s commitment toward universal screening.

## Introduction

Health systems have clear interest and new incentives to focus on patients’ social determinants of health (SDoH) due to growing evidence that reducing inequities in health outcomes depends on addressing an individual’s social needs [1]. The World Health Organization (WHO) defines SDoH as “the conditions, in which people are born, grow, work, live, and age, and the wider set of forces and systems shaping the conditions of daily life” [2]. These determinants drive inequity in health outcomes through the insidious effects of poverty and racism manifested through health-related social needs (HRSNs) such as limited access to nutritious foods, unemployment, and unstable, unaffordable, or low-quality housing [1]. According to the WHO Conceptual Framework for Action on the Social Determinants of Health, a health system is uniquely positioned to mitigate the effects of HRSNs by increasing access to and promoting integration of social care services.

Evidence has shown that unmet HRSNs contribute to poor health outcomes through increased exposure to risk factors for chronic conditions, higher likelihood of chronic stress, and decreased access to resources for those with preexisting conditions [3]. Patients with HRSNs also have higher emergency department utilization [4–6], higher hospital admissions [7,8], higher rates of hospital readmission [9], and higher rates of missed ambulatory appointments [10,11], which coincide with higher cost to the health system [12]. Recent interventions integrating social care in clinical settings have demonstrated improvements in health outcomes and cost by addressing food security [13], housing stability [14,15], and legal assistance [16,17].

In addition to health system factors driving HRSN screening, the regulatory context has shifted with the release of new health equity measures from the Centers for Medicare & Medicaid Services [18], the Joint Commission [19], and the National Committee for Quality Assurance [20]. The National Academies of Sciences, Engineering, and Medicine also recently provided health system guidance on HRSNs through the identification of five complementary activities, namely Awareness, Adjustment, Assistance, Alignment, and Advocacy, recommended to facilitate social care integration [21]. *Awareness* activities are intended to identify HRSNs and community assets; *Adjustment* aims to change the approach to clinical care to accommodate HRSNs; *Assistance* reduces the burden of HRSNs through social service navigation; *Alignment* invests in and facilitates the organizing of existing community assets to address HRSNs; and *Advocacy* promotes policies that facilitate the creation or redeployment of resources to address HRSN. Although this cascade of social care integration activities relies on efforts to increase *Awareness* of HRSNs, there are many practical challenges related to health systems’ ability to scale *Awareness* activities. This study describes the experience of one large urban health system in scaling HRSN Awareness efforts through screening and implementing improvement strategies over five years, from 2018 to 2023.

## Materials & methods

### Setting

In 2017, a multidisciplinary team of administrators, clinicians, social workers, and community-based partners was formed to develop a system-wide strategy to implement HRSN screening across a network of ambulatory and inpatient practices within a large, urban health system in Bronx County, New York [22]. The HRSN screening tool was initially tested for feasibility and acceptability at selected practices prior to its full-scale integration within the electronic health record (EHR) in April 2018. The standardized screening tool was adapted from a widely used, validated instrument, the Health Leads screening toolkit [23]. The final tool was launched across the health system, including inpatient, ambulatory primary care, and specialty practices, and included 10 HRSN categories: housing security, housing quality, food security, utilities, health transportation, medications, child or elderly care, legal services, family stress, and safety. The tool was designed to be self-administered and distributed during routine clinical visits in the nine most common languages in the catchment area. Data entry into the EHR was facilitated by standardized workflows that included both administrative and nursing staff members.

The screening tool was available to all clinical practices in the health system through EHR integration. Each practice had the discretion to select which patients should be screened and at what frequency based on patient volume and staff availability, given the lack of evidence-based guidelines. There was also variability in resources available at each practice with some clinical teams having full or part-time social workers or Community Health Workers (CHWs) to connect patients to essential social services, while others relied on resource lists generated from available social service resource directories. All practices, however, adhered to the core components of the intervention, which included using the standardized screening tool, providing patients with resources if a HRSN was identified and assistance was requested, and data entry in the EHR interface prior to the clinician visit. The study was reviewed and approved by the Albert Einstein College of Medicine institutional review board (2017-8434).

### Deliberate implementation strategies

We retrospectively reviewed implementation strategies utilized during the study period with key stakeholders and categorized each strategy according to the Expert Recommendations for Implementing Change (ERIC) implementation strategy taxonomy [24]. The ERIC taxonomy provides a uniform language for implementation strategies across contexts and clarity for separate and concrete actions. Use of a common language for implementation strategies in clinical and translational research supports efforts to implement and scale programs to other contexts. We selected the ERIC taxonomy for this study because of its fit in the healthcare context and recognition in the field [25,26].

The implementation strategies employed were deliberate attempts to increase the volume of HRSN screens administered within specific clinical teams and, more broadly, systematically across the health system. We did not consider events external to the health system (i.e., public health emergencies, state or national policy changes) in this analysis because, although they may have impacted screening, these events were not implemented as part of ongoing scale or quality improvement processes.

Implementation strategies included: (1) developing a novel role in the health system and appointing the first Director of SDoH to coordinate and support screening (January 2020), (2) creating an Executive Working Group consisting of key health system leaders (February 2021), (3) launching a Clinician Champion Working Group to foster a collaborative learning environment and support clinical teams that are directly implementing the intervention (April 2021), (4) disseminating a Screening and Referral Toolkit to provide centralized guidance and resources to assist clinical teams with effectively implementing the screening initiative (May 2021), (5) integrating the screening tool within the EHR supported online patient portal (June 2021), (6) expanding implementation to discrete clinical teams with leadership support (March 2022), (7) providing data feedback loops to track process measures with clinician champions and other stakeholders (September 2022), and (8) deploying CHWs to address HRSNs identified through screening and connect patients to social services (September 2022).

In most cases, implementation strategies were defined and planned several months prior to the described date of implementation. This pre-implementation process included changes in infrastructure, information technology development, networking and meeting with key stakeholders, and staff training. We reported a one-month time window (i.e., date of implementation) for each implementation strategy to represent the date when the strategy was first deployed or launched within the health system (e.g., first meeting for Executive Working Group, first time the Screening and Referral Toolkit was shared with clinician champions, first time screening was administered in the online patient portal).

**Figure 1.**
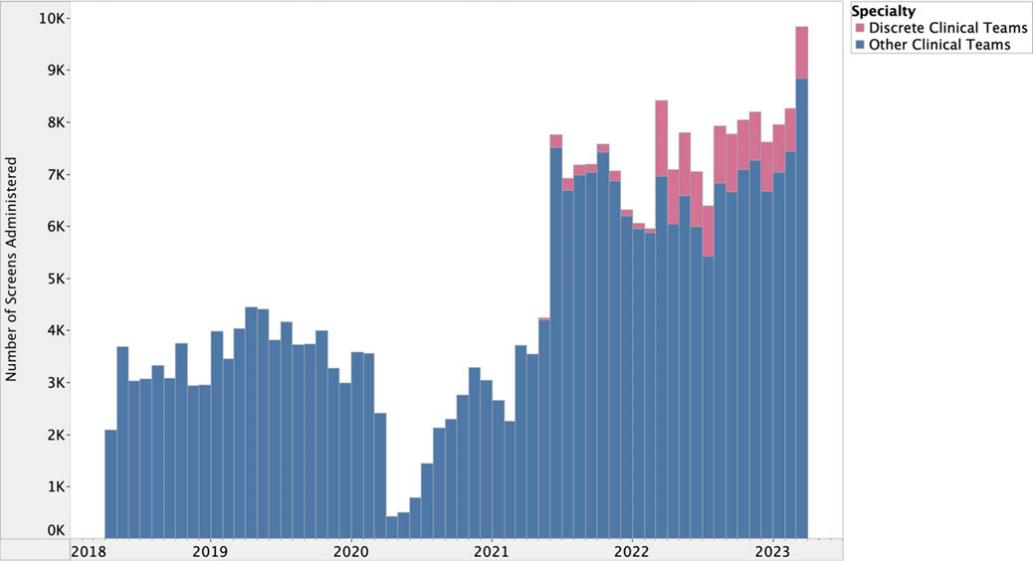
Prospective data review of expansion to discrete clinical teams, March 2022–March 2023.

**Figure 2.**
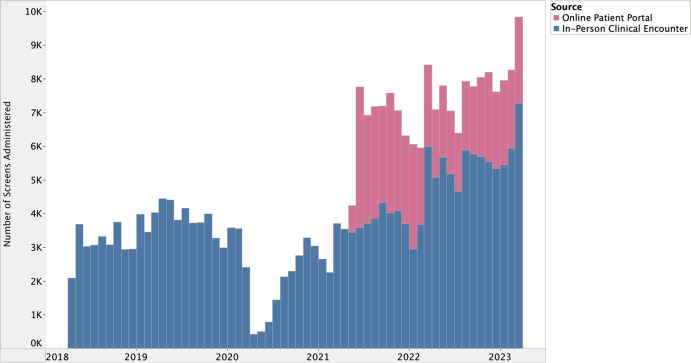
Prospective data review of integration into electronic health record supported online patient portal, June 2021–March 2023.

There is a well-defined lag between health research evidence generation and translation into clinical practice [27], however, the potential lag between implementation and effect is less clear in quality improvement processes. We hypothesized that many of our implementation strategies (#1–4 and #7–8) would have lagged (or potentially combined) effects on HRSN screening due to their reliance on behavior change, which includes adopting new roles, influencing other clinicians to screen for HRSNs, and utilizing toolkits and data feedback loops. Meanwhile, the date of implementation for strategies #5 and #6 reflects the date of effectuation due to prospective data review and validation in the EHR during the implementation period (Figs. 1 and 2).

### Statistical analysis

Measures related to the number of HRSN screens completed were extracted from the EHR using Microsoft SQL Server, version 18, to query data from the Epic Electronic Health Record Data Warehouse. The primary outcome of interest was the number of HRSN screens completed per month, which included data from patients screened multiple times across the study period.

We utilized statistical process control (SPC) methods to assess whether our implementation strategies contributed to special causes of variation over the first five years of the intervention [28,29]. The Individual and Moving Range (I-MR) Chart was selected to visualize the aggregate number of screens administered per month as well as the moving range, or the difference in screens between the current month and the previous month. The primary assumption of SPC is that all processes are subject to variation (i.e., common cause variation), which represents the random distribution of observations around the central tendency due to chance and inherent to the process.

All observations between the lower (LCL) and upper control limits (UCL), which are 3 sigmas below (μ – 3 σ) and above the mean, (μ + 3 σ) number of screens administered per month, respectively, were reported as common causes of variation [28]. Meanwhile, observations outside the LCL and UCL were defined as special cause variation. In addition to special cause variation, we also identified trends, defined as six consecutive points increasing or decreasing, in the number of screens administered over time. Measures in the I-MR Chart followed a normal distribution and were calculated using the SHEWHART procedure in SAS version 9.4 [30].

## Results

### Study sample

There were 280,757 total HRSN screens successfully administered across 311 distinct clinical teams in the health system during the first five years of implementation between April 2018 and March 2023. This represents an acceptance rate of 91.7%, with an additional 25,383 screens declined among 18,336 unique patients. Of the patients who declined a screen, 7,908 were excluded from the study sample and 10,428 were included for a screen accepted during another clinical encounter in the study period. The study sample includes data from 171,896 unique patients, of which 59,914 (34.9%) were successfully screened multiple times.

The HRSN screening tool was primarily administered in outpatient settings (91.6%) with few screens completed in inpatient and emergency settings (8.4%). There were 101,738 (39.6%) screens administered in outpatient internal medicine, 83,935 (32.6%) in pediatrics, 40,307 (15.7%) in family medicine, 14,939 (5.8%) in obstetrics/gynecology, 10,795 (4.2%) in the care management organization, 1,275 (0.5%) in cancer care, and 4,168 (1.6%) screens administered in other outpatient programs.

**Figure 3.**
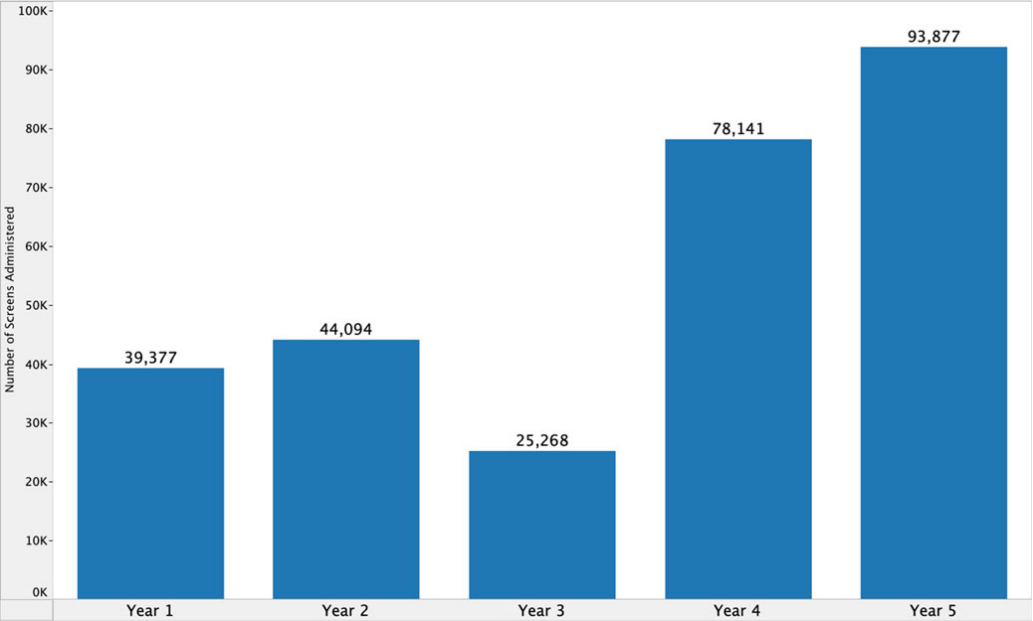
Screens administered per year of health-related social need screening program, April 2018–March 2023.

Additional descriptive statistics are included to better understand the reach of implementation but are limited to outpatient practices with documented HRSN screens during the study period. Between April 2018 and March 2023, the HRSN screening tool was administered at 5.1% (*n* = 257,157) of clinical encounters (*N* = 5,075,308) in outpatient practices of interest. Meanwhile, 25.7% (*n* = 154,651) of active patients (*N* = 602,780) were screened for HRSNs. In the first year of implementation, 39,377 screens (3.9% of 1,012,094 encounters) were administered to 34,003 unique patients (10.2% of 334,278 active patients) across 90 clinical teams (Fig. 3). By the fifth year, 93,877 screens (9.1% of 1,033,860 encounters) were administered to 80,527 unique patients (25.4% of 316,431 active patients) across 223 clinical teams.

Of the total 171,896 unique patients screened, most were between 30 and 64 years of age (40.9%), female (62.0%), Hispanic (39.2%) or non-Hispanic Black (29.2%), preferred English as their primary language (82.1%), and enrolled in Medicaid (39.8%) or commercial insurance (32.8%), according to their most recent screen (Table 1). High HRSN screening completion rates for female patients reflect the higher distribution of females in the active outpatient population (59.6%) as well as utilization of the screening tool in obstetrics/gynecology practices (5.8% of screens). There were 26,193 patients (15.2%) who reported at least one HRSN, with food security (4.9%), housing quality (4.6%), housing security (4.0%), and healthcare transportation (3.6%) identified as the most frequently reported HRSNs (Table 2).

**Table 1.** Descriptive characteristics of unique patients screened for health-related social needs within a large urban health system, 2018–2023

	N (%)
Total unique patients screened	171,896 (100.0%)
Age at most recent screening	
0–5	17,464 (10.2%)
6–11	15,183 (8.8%)
12–19	16,857 (9.8%)
20–24	9,269 (5.4%)
25–29	9,214 (5.4%)
30–64	70,377 (40.9%)
65+	33,532 (19.5%)
Sex	
Male	65,354 (38.0%)
Female	106,521 (62.0%)
Unknown	21 (0.01%)
Race and ethnicity	
Non-Hispanic White	17,784 (10.4%)
Hispanic	67,357 (39.2%)
Non-Hispanic Black	50,142 (29.2%)
Non-Hispanic Asian/Pacific Islander	4,518 (2.6%)
Non-Hispanic American Indian/Alaskan Native	684 (0.4%)
Other	13,850 (8.1%)
Declined to report or not available	17,561 (10.2%)
Preferred language	
English	141,129 (82.1%)
Spanish	25,118 (14.6%)
Other	3,999 (2.3%)
Declined to report or not available	1,650 (1.0%)
Insurance status	
Commercial	56,380 (32.8%)
Medicaid	68,416 (39.8%)
Medicare	29,655 (17.3%)
Other	7,107 (4.1%)
Uninsured	10,338 (6.0%)

**Table 2.** Health-related social need status of unique patients screened within a large urban health system, 2018–2023

	N (%)	
Total unique patients screened	171,896 (100.0%)	
	Yes, *n* (%)	No/Declined to Report, *n* (%)
At least 1 HSRN at most recent screening	26,193 (15.2%)	145,703 (84.76%)
Housing security	6,922 (4.1%)	164,974 (95.97%)
Housing quality	7,932 (4.6%)	163,964 (95.39%)
Food security	8,395 (4.9%)	167,081 (97.20%)
Utilities	4,815 (2.8%)	163,501 (95.12%)
Health transportation	6,191 (3.6%)	165,705 (96.40%)
Medications	5,166 (3.0%)	166,730 (96.99%)
Child or elderly care	3,905 (2.3%)	167,991 (97.73%)
Legal services	4,135 (2.4%)	167,761 (97.59%)
Family stress	3,891 (2.3%)	168,005 (97.74%)
Safety	1,240 (0.7%)	170,656 (99.28%)

### Implementation strategies & statistical process control

**Figure 4.**
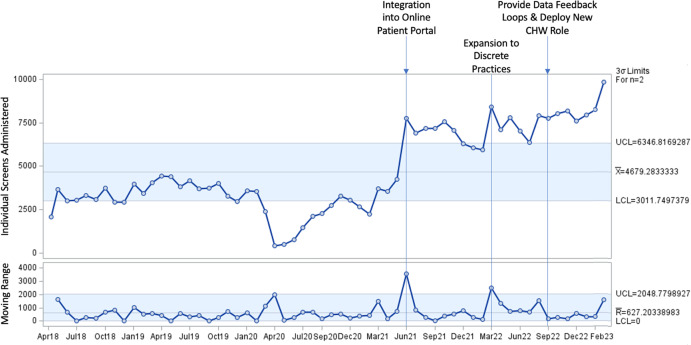
Individual-moving range chart of health-related social need screens administered per month, April 2018–March 2023.

Figure 4 presents I and MR Charts of screening data over time overlayed with retrospectively reviewed implementation strategies linked to abnormal or special cause variation. In the first five years of implementation, 35 of the 60 months (58.3%) signaled abnormal variation (Fig. 4). Special cause variation in the I Chart (below the LCL = 3,011.75) was first detected in November 2019, December 2019, and December 2020; however, these observations did not coincide with known changes to implementation strategies (Table 3).

**Table 3.** Deliberate implementation strategies & expert recommendations for implementing change (ERIC) categories [24]

Deliberate implementation strategy	Description of deliberate implementation strategy	ERIC implementation strategy	ERIC implementation strategy definition
1: Appointed “Director of Social Determinants of Health” in the health system (January 2020)	Created a new leadership position to facilitate uptake of social needs screening across the health system	Mandate change	Have leadership declare the priority of the intervention and their determination to have it implemented
2: Launched Executive Working Group for HRSN screening (February 2021)	Developed working group with key members of health system leadership and influential colleagues and organized monthly meetings to inform implementation and influence colleagues to adopt screening initiative	Involve executive boards	Involve existing governing structures (*e.g.*, boards of directors, medical staff boards of governance) in the implementation effort, including the review of data on implementation processes
3: Launched Clinician Champion Working Group for HRSN screening (April 2021)	Developed working group with clinicians who are implementing the screening initiative and organized monthly meetings to foster a collaborative learning environment to share barriers and facilitators to implementation	Organize clinician implementation team meetings	Develop and support teams of clinicians who are implementing the innovation and give them protected time to reflect on the implementation effort, share lessons learned, and support one another’s learning
4: Developed & Disseminated HRSN Screening and Referral Toolkit to clinical partners (May 2021)	Developed and distributed a toolkit to provide centralized guidance and resources to assist clinical practices with effectively implementing the screening initiative	Develop & distribute educational materials	Develop and distribute manuals, toolkits, and other supporting materials in ways that make it easier for stakeholders to learn about the innovation and for clinicians to learn how to deliver the clinical innovation
5: Integrated HRSN screening tool into the EHR supported online patient portal (June 2021)	Launched HRSN screener in new platform, the online patient portal, which allowed patients to self-administer the screen online prior to their scheduled clinical appointment	Involve patients/consumers and family members	Engage or include patients/consumers and families in the implementation effort
6: Expanded social needs screening to discrete clinical practices (March 2022)	Launched implementation tool in new clinical practices with full leadership support	Change service sites	Change the location of clinical practices to increase access
7: Provided data feedback loops to clinician champions and other stakeholders (September 2022)	Developed interactive dashboards with key implementation measures and shared with clinician champions, administrators, and leadership	Facilitate relay of clinical data to providers	Provide as close to real-time data as possible about key measures of process/outcomes using integrated modes/channels of communication in a way that promotes use of the targeted innovation
8: Deployed new role of “Community Health Worker” in the health system (September 2022)	Created new positions to connect patients who reported unmet HRSNs with essential social services	Create new clinical teams	Change who serves on the clinical team, adding different disciplines and different skills to make it more likely that the clinical innovation is delivered (or is more successfully delivered)

Between March 2020 and October 2020, we observed special cause variation (below LCL = 3,011.75) coinciding with the start of the COVID-19 pandemic. Between April 2020 and September 2020, we also witnessed a positive trend (i.e., six consecutive points increase) in screening, which indicates the return to baseline prior to COVID-19. Special cause variation below the LCL was also observed in January 2021 and February 2021 coinciding with the rapid emergence of the SARS-CoV-2 B.1.526 variant in New York City (NYC) [31].

The I chart first suggested special cause variation above the UCL (UCL = 6,346.82) between June 2021 and November 2021 followed by another period of sustained variation between March 2022 and March 2023. Special cause variation identified in June 2021 coincided with the integration of the HRSN screening tool into the EHR’s online patient portal (Fig. 1). This special cause variation (above UCL = 6,346.82) was sustained through November 2021.

The second period of special cause variation above the UCL in the I chart aligned with the systematic launch of the intervention across all ambulatory practices within one clinical department in March 2022 (Fig. 2). Although special cause variation was sustained above the UCL through March 2023, there appears to be an increase in screening between July and August 2022. This is likely due to the anticipation of CHWs deployed in September 2022, which required clinical teams to screen patients before completing referrals to address unmet HRSNs. The deployment of CHWs was implemented alongside the launch of data feedback loops, which were focused on implementation outcomes and measures tracking successful connections to social services. These strategies likely contributed cumulative effects on HRSN screening volume over the remainder of the study period.

The MR chart detected two timepoints with special cause variation above the UCL (UCL = 2,048.78), first in June 2021 and then in March 2022, which support the previously described associations between screening and integration into the online patient portal and expansion to clinical teams, respectively. These implementation strategies yielded the greatest impact on screening.

## Discussion

We determined that scaling HRSN screening within a large health system over time is feasible and can be accelerated through targeted strategies that foster screening activities. In our health system, the volume of HRSN screening increased nearly three-fold from year one to year five of implementation. We observed multiple implementation strategies that seemed to influence screening including the deployment of CHWs to connect patients with HRSNs to social services and the provision of data feedback loops to track key process measures; however, the integration of the screening tool into the EHR’s online patient portal and deliberate expansion within a clinical department yielded the largest observed increases in HRSN screening. The sustained increase in screening observed over the last two years of implementation, even in the wake of COVID-19, was likely due to the cumulative effects of the implementation strategies cited and the health system’s continued commitment to improving screening.

Several studies have evaluated facilitators and barriers to screening for HRSNs in clinical settings; however, the most relevant to our study is that from the NYC Health + Hospitals (H + H) SDoH screening and referral program [32]. The challenges identified by NYC H + H include integration of screening into clinical workflows, burden on staff, limitations within the patient population (e.g., health literacy, English proficiency, immigration status, screening fatigue), and capacity for data entry. NYC H + H also defined best strategies utilized in their intervention including the unique adaptation of the screening tool for each practice, integration of screening into existing workflows, promotion of universal screening, development of referral resources, utilization of technology and data systems, and allocation of dedicated staff for the intervention. Most of the previously described best practices align with implementation strategies applied in our intervention except for the adaptation of the screening tool, which is standardized across our health system.

Although NYC H + H integrated the HRSN screening tool into the EHR, there was no mention of its administration in the health system’s online patient portal. In our study, we hypothesize that integrating the screening tool into the patient portal expanded the reach of the intervention by automating the screening process for patients enrolled with an upcoming clinical visit. Several studies have reported staff concerns with time needed to complete HRSN screeners [33–35]. Clinical teams within our health system have expressed that integration into the patient portal has reduced the additional burden on staff, who were previously solely responsible for administering screens and documenting patient responses. The automation of HRSN screening in patient portals has demonstrated success in other studies [36,37] with some patients reporting a greater likelihood to endorse HRSNs in asynchronous modalities [38]. This strategy, however, has also been identified as a barrier in clinical practices where enrollment is low [39].

Integrating the screening tool into the patient portal increases patient engagement with implementation, but there remains a demonstrated need to improve clinician motivation to screen and capacity to act on HRSNs identified. Clinicians have previously reported both a lack of training on HRSNs and lack of resources to address HRSNs as key barriers to screening [34,40–42], with some arguing that screening for HRSNs without linking patients to resources is ineffective and unethical [43]. In addition to clinicians, patients have also expressed the importance of clarifying the purpose of the HRSN screen, especially if to connect patients to social services [38,42]. CHWs have been proven to be an effective way to address HRSNs in several studies, including within our health system [44]. Addressing HRSNs through CHWs and facilitating the relay of CHW referral data can increase screening behavior among clinical teams.

Expansion of HRSN screening to targeted, new clinical teams across the health system is another key facilitator to screening interventions [32,45]. Our health system recommends but does not mandate universal screening for HRSNs; therefore, as seen in our analysis, continuing to expand implementation to discrete clinical practices is critical to increasing the reach of the intervention. Although all clinical teams have access to the screening tool in the EHR, aligning around a universal health system target to foster accountability at each practice and specialty may further advance screening.

### Limitations

There are several limitations to address in this study. First, we retrospectively reviewed implementation strategies and their dates of implementation, which were selected based on staff recall and available documentation. This may introduce both recall and selection bias, as strategies may have been selected by stakeholders based on their perceived significance. We also linked implementation strategies in this study to changes in HRSN screening rates within a one-month period (i.e., within the defined “implementation window”). This limits our ability to determine the lagged and combined effects of implementation strategies over time. It may also explain the observed significance of implementation strategies with hypothesized immediate effects while undervaluing the effects of implementation strategies, which may be lagged, or those whose effects were most pronounced in combination with other strategies. There is limited evidence on the lag time required for clinicians and stakeholders to change their behaviors, which overall limits our interpretation of the results [46].

We only selected implementation strategies utilized at the system level, not the clinical team level; therefore, we cannot conclude whether special cause variation was signaled from only one or a subset of clinical teams. We also cannot account for all external events that may have impacted screening in this analysis. Regarding COVID-19, we could not connect screening data with surges within the health system specifically, only across the Bronx and NYC.

For both implementation strategies and external events, we cannot assume causality for variations and trends in screening identified through SPC methods. Additionally, since SPC methods were not initially developed for health research, the control chart cannot account for provider or patient-level variability in screening behavior. In the SPC analysis, we also did not account for the difference in the number of active patients or completed clinical visits per month. There are potential autocorrelation issues given the cumulative effects of implementation strategies on performance improvement, with previous increases in screening predicting future increases in screening.

Another potential limitation of this study is that the sample of patients screened may not be representative of all active patients in the health system. Clinical teams had the discretion to screen subsets of their patient population, with some only screening new patients, patients with scheduled annual visits, or patients assumed to have HRSNs. While we are unable to define if the subset of screened patients is categorically different from those that have gone unscreened, we can say that the demographic characteristics of screened patients match those of the overall health system.

## Conclusions

Integrating a learning health system approach that identifies strategies that foster HRSN screening uptake offers a path forward to promoting health equity activities. We found that implementation strategies designed to promote efficiency, foster universal screening, link patients to resources, and provide clinical teams with an easy-to-integrate tool seem to have the greatest impact on the yield of completed HRSN screeners. More rigorous research, including mixed methods studies, is still needed to advance practice-based evidence that will promote health equity within our health systems and integrate comprehensive clinical and social care for our patients.
